# Enhanced Superconductivity near the Pressure-Tuned Quantum Critical Point of Charge-Density-Wave Order in Cu_1-δ_Te (δ = 0.016)

**DOI:** 10.3390/ma18215042

**Published:** 2025-11-05

**Authors:** Kwang-Tak Kim, Yeahan Sur, Ingyu Choi, Zifan Wang, Sangjin Kim, Dilip Bhoi, Duck Young Kim, Kee Hoon Kim

**Affiliations:** 1 Center for Novel States of Complex Materials Research, Department of Physics and Astronomy, Seoul National University, Seoul 08826, Republic of Korea; 2 Center for High Pressure Science and Technology Advanced Research (HPSTAR), Shanghai 201203, China; zifan.wang@hpstar.ac.cn; 3 Institute of Applied Physics, Department of Physics and Astronomy, Seoul National University, Seoul 08826, Republic of Korea

**Keywords:** charge density wave, quantum critical point, superconductivity, low-dimensional materials, high-pressure

## Abstract

We have investigated the evolution of CDW states and structural phases in a Cu-deficient Cu_1-δ_Te (δ = 0.016) by employing high-pressure experiments and first-principles calculations. Raman scattering results reveal that the vulcanite structure at ambient pressure starts to change into the Cu-deficient rickardite (*r*-CuTe) structure from 6.7 GPa, which then becomes fully stabilized above 8.3 GPa. Resistivity data show that *T*_CDW1_ (≈333 K) is systematically suppressed under high pressure, reaching zero at 5.9 GPa. In the pressure range of 5.2–8.2 GPa, a sharp resistivity drop due to superconductivity occurs at the onset temperature *T*_C_ = ~2.0–3.2 K. The maximum *T*_C_ = 3.2 K achieved at 5.6 GPa is clearly higher than that of CuTe (2.3 K), suggesting the importance of charge fluctuation in the vicinity of CDW suppression. At 7.5 GPa, another resistivity anomaly appears due to the emergence of a second CDW (CDW2) ordering at *T*_CDW2_ = ~176 K, which exhibits a gradual increase to ~203 K with pressure increase up to 11.3 GPa. First-principles calculations on the Cu-deficient Cu_11_Te_12_ with the *r*-CuTe structure show that including on-site Coulomb repulsion is essential for incurring an unstable phonon mode relevant for stabilizing the CDW2 order. These results point out the important role of charge fluctuation in optimizing the pressure-induced superconductivity and that of Coulomb interaction in creating the competing CDW order in the Cu-deficient CuTe system.

## 1. Introduction

Low-dimensional materials exhibiting charge density wave (CDW) orders [1,2,3,4,5,6,7,8,9] have attracted significant attention due to their rich phase diagrams, including superconductivity (SC) [1,4], nematicity [5], and multiple density-wave orders [4,5,6,7,8]. Suppression of CDW by external parameters in those low-dimensional materials often leads to emergent novel quantum states, making them an ideal platform for exploring the interplay between the CDW and the competing quantum states. Archetypal examples include the case of transition-metal dichalcogenides Pd*_x_*TaSe_2_, in which intercalating Pd ions between TaSe_2_ layers suppresses the CDW and enhances SC near the CDW quantum critical point [1], and that of the kagome metals AV_3_Sb_5_ (A = K, Rb, Cs), at which the pressure-induced suppression of the CDW state results in two SC domes [8,9]

CuTe represents a prototypical example of exhibiting CDW order from the quasi-one-dimensional (Q1D) electronic structure. Angle-resolved photoemission spectroscopy has confirmed that the CDW transition appearing at *T*_CDW1_ = 335 K at ambient pressure is driven by the Fermi surface nesting of Te 5*p_x_* orbitals [10]. Under high-pressure conditions [11,12], CuTe exhibits a complex pressure–temperature phase diagram involving an interplay between CDW and SC phases (Figure S1). As pressure increases, *T*_CDW1_, as evidenced by a jump in resistivity*,* decreases linearly and reaches ~100 K near 6.5 GPa. Above this pressure, the anomaly vanishes. Beyond ~6.7 GPa, another in-plane resistivity anomaly—a peak in the *dρ*/*dT* curve—appears at ~200 K, indicating a stabilization of a second CDW phase (CDW2). With further pressure (6.7 ≤ *P* ≤ 10 GPa), *T*_CDW2_ is gradually lowered toward ~170 K, and the resistivity anomaly at *T*_CDW2_ finally vanishes above 10 GPa. Moreover, the SC first emerges at a pressure of 4.8 GPa as a small resistivity drop at *T*_C_ ≈ 0.5 K [12]. *T*_C_ rises with further increase in pressure, showing a maximum at 2.3 K near 6.5 GPa. Above 6.5 GPa, *T*_C_ gradually decreases, consequently forming a dome-like phase diagram. Beyond 10 GPa, *T*_C_ becomes below 1 K, indicating significant suppression of SC with increasing pressure. At pressures exceeding 20 GPa, CuTe undergoes a structural phase transition from the orthorhombic (*Pmmn*) to the monoclinic (*Cm*) phase. In this new structural phase, SC is stabilized with *T*_C_ ≈ 2.4 K, persisting across the high-pressure range up to 49 GPa [11].

However, the nature of pressure-induced SC in CuTe, particularly its relationship with the CDW orders, remains elusive. As a possible mechanism for finding SC in the vicinity of CDW phases, two scenarios have been proposed [12]. First, a continuous suppression of CDW1 may increase charge fluctuation to enhance the pairing strength for superconductivity. However, this scenario is not applicable to the pristine CuTe as *T*_CDW1_ decreases to only 100 K at 6.5 GPa, thus mitigating the possibility of having quantum fluctuation of the CDW order parameters. Second, a nominal competition between CDW and SC may induce the stabilization of SC at the expense of the competing CDW order. As is common in a quasi-1D CDW system, the charge density modulation seems to involve significant lattice distortion so that the CDW transition with variation in temperature or pressure is clearly a first-order type. In particular, as a function of pressure, an abrupt first-order transition from CDW1 to CDW2 occurs at ~6.5–6.7 GPa, so that SC is not a single competing phase of the CDW1 order. Indeed, SC stabilized at 4.8 ≤ *P* ≤ 10 GPa also overlaps with the temperature and pressure windows where CDW2 phase stabilized (i.e., 7.5 GPa (*T*_CDW2_ = 204 K) ≤ *P* ≤ 10.1 GPa (*T*_CDW2_ = 173 K)). Therefore, the SC appears within the electronic structure created by the CDW2 phase rather than appearing directly at the expense of the CDW1 phase.

To address the puzzling question on the relationship between pressure-induced SC with the CDW order, we have investigated a Cu_1-δ_Te (δ = 0.016) single crystal using high-pressure studies of Raman spectroscopy, transport, and structural properties. Our study reveals that slight Cu deficiency significantly modifies the phase evolution, leading to *T*_CDW1_ ≈ 0 K near 6 GPa and subsequent emergence of SC above 5.2 GPa with the maximum *T*_C_~3.2 K at 5.6 GPa. Furthermore, we find that a new structural phase, isostructural to the Cu-deficient rickardite CuTe (*r*-CuTe), appears above 6 GPa, thereby hosting the CDW2 order around 200 K. These findings suggest that understanding the role of Cu deficiency can be crucial for unraveling the intricate relationship between structural transitions, CDW orders, and superconductivity in the copper telluride system.

## 2. Methods

### 2.1. Single-Crystal Growth and Characterization

Cu_1-d_Te (d = 0.016) single crystals were synthesized by a Te self-flux method. High-purity Cu powder (99.95%, Alfa aesar, Ward Hill, MA, USA) and Te chunks (99.9999%, Alfa aesar) were mixed in a molar ratio of 1:2. The mixture was placed in an alumina crucible, which was then covered with another alumina crucible filled with quartz wool to enable filtration of the crystals from the Te flux after centrifugation. All the preparations were carried out in an Ar gas environment inside a glove box, of which oxygen and moisture concentrations were maintained below 1 ppm. The crucibles were subsequently sealed in evacuated quartz tubes. The ampules were heated to 660 °C and held at that temperature for 24 h before being cooled down to 400 °C at a rate of 1 °C/h. Centrifugation was employed to separate the crystals from the Te flux, resulting in plate-like crystals with gold-colored surfaces. A typical size of the crystals was ~3 × 3 × 0.05 mm^3^. Room-temperature X-ray diffraction (XRD) measurements were performed using a high-resolution X-ray diffractometer (Empyrean^TM^, PANalytical, Worcesterchire, UK). To characterize the stoichiometry of the Cu_1-δ_Te (δ = 0.016), wavelength-dispersive X-ray spectroscopy was performed in a field emission electron probe microanalyzer (JEOL Ltd., Tokyo, Japan, A-8530F), installed at the National Center for Inter-university Research Facilities (NCIRF) at Seoul National University; Cu (99.8%) and Te (99.9999%) metals were used as standard specimens.

### 2.2. High-Pressure Transport and Raman Measurements

Longitudinal resistivity along the ***a***-axis and transverse Hall resistivity were measured by use of two lock-in amplifiers (SR830) and a standard Hall bar method. Temperature *T* and external magnetic fields *H* were controlled using a Physical Property Measurement System (PPMS^TM^, Quantum Design, San Diego, CA, USA). Pressure was applied using a diamond anvil cell attached with a pair of diamonds of a 300 μm culet size; sodium chloride (NaCl) powder was used as a pressure-transmitting medium, and c-BN as the insulating layer. Raman measurements were performed using diamond anvil cells equipped with diamond anvils of a 300 μm culet size. Raman measurements at room temperature were conducted using a 532 nm laser beam, and finely ground NaCl served as a pressure-transmitting medium. As summarized in Figure S2, the intensity of the laser less than 1 mW did not induce the phase decomposition, even though we increased the time to measure. However, when we increased the intensity above 2 mW, the phase decomposition was observed. Based on these results, our measurements under pressure were performed with the laser, with an intensity of 0.5 mW, and we also accumulated the data for 1 min to reduce the possible decomposition.

### 2.3. First-Principles Phonon Calculations

Phonon band structures for Cu_11_Te_12_ in the *r*-CuTe structure were calculated using the finite displacements method to obtain the dynamical matrix with Vienna ab initio simulation package (VASP) [13,14] and PHONOPY [15]. We used the generalized gradient approximation (GGA) of Perdew–Burke–Ernzerhof (PBE) [16] for the exchange-correlation functional with the cut-off energy of 600 eV. Monkhorst–Pack *k*-point mesh with 0.03∙(2π/Å) and 0.04∙(2π/Å) mesh resolution was used for the structural optimization and phonon band structures calculation, respectively. For phonon calculations, we used the 2 × 2 × 2 supercell. We conducted the PBE + U calculations to account for the correlated *d* orbitals of Cu, and *U*_eff_ = *U* − *J* was chosen from 0 eV to 9 eV to investigate the Coulomb correlation effect on the structural instability. The effect of Van der Waals interaction was also considered using the DFT-D3 method with zero damping (D3) [17].

## 3. Results

- Crystallographic structure and basic physical properties of Cu_0.984_Te

**Figure 1:**
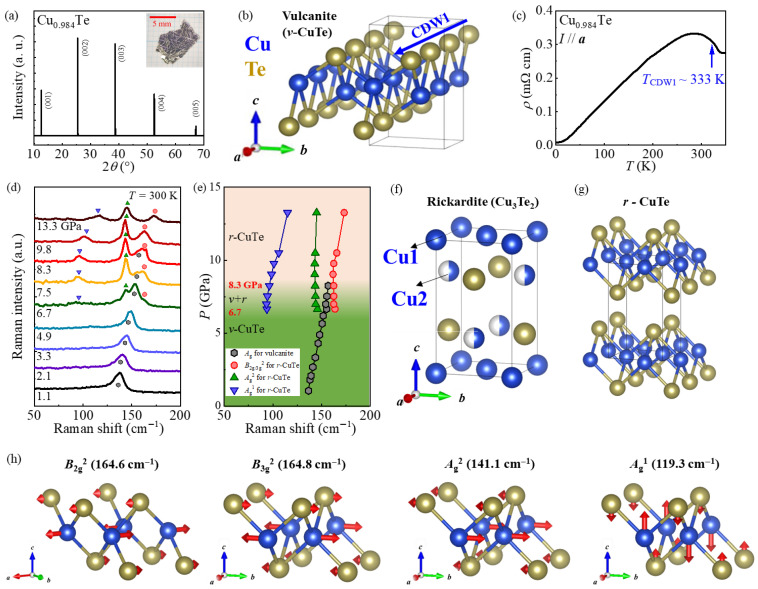
(**a**) The single-crystal X-ray diffraction pattern on the *ab*-plane of the single crystal Cu_0.984_Te at 300 K. The inset figure shows a picture of a typical Cu_0.984_Te single crystal by flux methods. (**b**) The crystal structure of the vulcanite CuTe (*v*-CuTe). The blue arrow represents a quasi-one-dimensional Te chain direction exhibiting CDW (CDW1) order along the ***a***-axis. (**c**) The temperature dependence of the *a*-axis resistivity ρ of a Cu_0.984_Te single crystal at ambient pressure. The blue arrow indicates the CDW1 ordering temperature (*T*_CDW1_ = 333 K). (**d**) The raw data obtained from high-pressure Raman spectroscopy measurements at 300 K in the applied pressure range from 1.1 to 13.3 GPa. The assigned Raman phonon modes are indicated by the symbols. (**e**) The variation in Raman mode frequencies with pressure. The symbols appearing in (**d**) were used to represent each Raman mode. (**f**) The crystallographic structures of the rickardite (Cu_3_Te_2_), reproduced from [18]. (**g**) The crystallographic structures of the Cu-deficient rickardite *r*-CuTe, reproduced from [18]. (**h**) The vibration related to the representative Raman phonon modes in the *r*-CuTe structure, reproduced from ref. [18].

Figure 1a presents an XRD pattern of a Cu_0.984_Te single crystal. Only sharp (001) diffraction peaks appear, indicating that the crystallographic ***c***-axis is perpendicular to the facet of the crystal. The measured (001) peaks match well with those of the vulcanite CuTe (*v*-CuTe, space group *Pmmn*). Independent XRD experiments on the grounded polycrystals have confirmed the *v*-CuTe structure (Figure S3). Therefore, the Cu_0.984_Te crystal is isostructural to the *v*-CuTe structure, as illustrated in Figure 1b, at ambient pressure and at room temperature. In the *v*-CuTe structure, the Te chains are known to exist along the *a*-axis, and each Cu atom forming a buckled Cu plane is bonded to the four Te atoms located below and above the Cu plane. As a result, the *v*-CuTe forms an orthorhombic *Pmmn* structure with lattice constants *a* = 3.16 Å, *b* = 4.08 Å, and *c* = 6.93 Å. Wavelength-dispersive X-ray spectroscopy confirmed a Cu:Te ratio of 0.984:1.00 (Figure S4, Table S1), indicating slight Cu deficiency. We also measured the resistivity along the *a*-axis, *ρ*, in a Cu_0.984_Te single crystal, as shown in Figure 1c. Despite the slight Cu deficiency, we observe a clear resistivity upturn near 333 K, a feature similar to CuTe at its CDW state [10,11].

B. Pressure-dependent evolution of Raman phonon modes

To monitor the structural evolution of Cu_0.984_Te, we performed high-pressure Raman scattering using diamond anvil cells up to 13.3 GPa at room temperature, as presented in Figure 1d. From the obtained scattering intensity, the pressure dependence of phonon mode frequencies was extracted as summarized in Figure 1e. Below 6.2 GPa, only one Raman mode was found at 136.6 cm^−1^, which seems to be close to a theoretically predicted frequency of the *A*_1g_ mode (136.0 cm^−1^) in the *v*-CuTe [11,19]. However, above 6.7 GPa, three new Raman modes, which do not correspond to the reported Raman modes of the *v*-CuTe [12,18], emerge at 163.8 cm^−1^(**mode 1**), 145.8 cm^−1^(**mode 2**), and 93.8 cm^−1^(**mode 3**). Meanwhile, the original *A*_1g_ mode persists up to 8.3 GPa and disappears above the pressure. These spectral changes provide evidence that a new structural phase appearing at 6.7 GPa coexists with the low-pressure *v*-CuTe up to 8.3 GPa and becomes solely stabilized above 8.3 GPa. Note that in the pristine CuTe, a pressure-induced structural transition has been reported to occur at 20 GPa from an orthorombic (*Pmmn*) to a monoclinic structure (*Cm*). However, no structural transition has been reported in a low-pressure regime around ~7 GPa [11,12].

One might suspect that the newly appearing modes 2 and 3 at the high pressures may have originated from Te clusters [19] as they are indeed close to those of the *E* modes in pristine Te at ambient pressure (140.7 cm^−1^ and 92.2 cm^−1^) [20,21]. However, if those modes originated from the Te clusters, the *A*_1_ mode of Te would have also been observed, since the intensity of the *A*_1_ mode is known to always be stronger than that of the *E* modes, and its frequency lies around ~120–100 cm^−1^ in a pressure regime below 15 GPa [20]. No signal that can be assigned as an *A*_1_ mode of Te was detected around ~100–120 cm^−1^ in our experiments performed below 13 GPa. The minimum frequency of the *A*_1_ mode of Te was previously found at ~100 cm^−1^ at a pressure of ~8 GPa [20]; its frequency at high pressures always remains higher than that of mode 3, leading to the conclusion that mode 3 is not the *A*_1_ mode of Te. Moreover, the Raman mode of Te has not been reported previously near the frequency region of ~164 cm^−1^ (that of mode 1) in a pressure window below 15 GPa, ruling out the possibility that mode 1 can stem from the possible Te cluster. These observations suggest that the three modes emerging under the pressure above 6.7 GPa are unlikely to originate from possible Te clusters.

According to the previous density functional theory (DFT) calculations on CuTe [20], it was predicted that the Cu-deficient rickardite structure (*r*-CuTe) would have a lower formation energy than the *v*-CuTe. In this theoretical *r*-CuTe structure plotted in Figure 1g [18,22], the Cu_2_ sites of the rickardite Cu_3_Te_2_ (Figure 1f) located close to the four Te sites become completely vacant, and the remaining Cu sites form a flat Cu plane. Although the *r*-CuTe forms an orthorhombic structure (space group *Pmmn*) with lattice constants *a* = 3.845 Å, *b* = 3.847 Å, and *c* = 6.493 Å, it is indeed close to a tetragonal structure as it has similar in-plane lattice constants. However, this theoretical *r*-CuTe phase has not been found experimentally in the high-pressure region of CuTe [11,12].

We find in Figure 1d that three Raman modes appearing at the high pressures above 6.7 GPa match relatively well with those predicted for the *r*-CuTe in ref. [18]; the phonon frequencies of mode 1 (163.8 cm^−1^) and mode 2 (145.8 cm^−1^) are close to those calculated for the *B*_2g_^2^/*B*_3g_^2^ (~164.7 cm^−1^) and the *A*_g_^2^ (141.1 cm^−1^) modes, respectively. According to Figure 1e, the mode 3 frequency shows a drastic increase with pressure from 93.8 cm^−1^ at 6.7 GPa to 115.0 cm^−1^ at 13.3 GPa. As a result, the predicted *A*_g_^1^ mode frequency (~119.28 cm^−1^) [18] is indeed close to the frequency of 115.0 cm^−1^ obtained at 13.3 GPa. As this *A*_g_^1^ mode involves the vibrational motion along the ***c***-axis, its frequency can be sensitive to the interlayer distance; consequently, mode 3 indeed exhibits drastic hardening under pressure. Therefore, mode 3 can be assigned as the *A*_g_^1^ mode predicted in the *r*-CuTe structure. On the other hand, we could not identify the additional phonon modes near the theoretically predicted *B*_2g_^1^/*B*_3g_^1^ mode (78.4 cm^−1^) within our experimental resolution.

C. Temperature-dependent resistivity under high pressures

**Figure 2:**
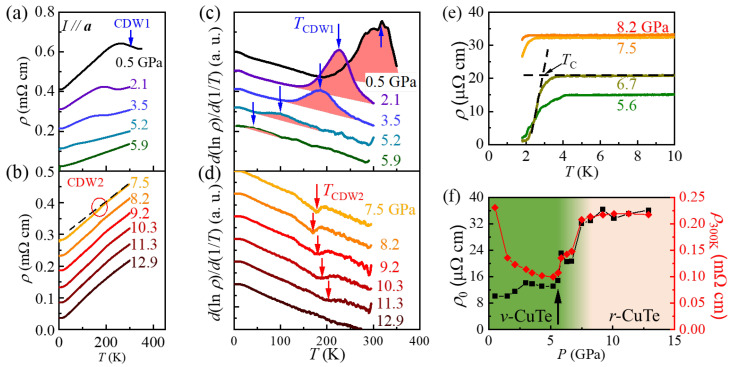
(**a**) Resistivity along the ***a***-axis (*r*) of a Cu_0.984_Te crystal at the pressure *P* below 5.9 GPa, exhibiting *ρ* increase due to the CDW1 (a blue arrow). The data is shifted down by a constant value (0.1 mΩ cm) for clarity. (**b**) *ρ* curves of Cu_0.984_Te at *P* above 5.9 GPa. A red circle highlights a region with small resistivity anomalies caused by the CDW2 phase, of which the derivative is plotted in (**d**). The data is shifted down by a constant value (0.05 mΩ cm) for clarity. (**c**) The *d* (ln *ρ*)/*d* (1/T) vs. T plots at *P* ≤ 5.9 GPa. The blue arrows indicate the peaks in each plot, representing *T*_CDW1_. (**d**) The *d* (ln *ρ*)/*d* (1/*T*) plots at *P* ≥ 7.5 GPa. The red arrows indicate the peaks due to stabilization of the CDW2 phase in the new *r*-CuTe structure. Both data in (**c**,**d**) are shifted by a constant value for clarity. (**e**) *ρ* of Cu_0.984_Te under pressure at low temperatures exhibits a sharp drop due to the onset of the superconducting transition. (**f**) The pressure-dependent variation in residual resistivity (*ρ*_0_) and resistivity at 300 K (*ρ*_300K_). Both *ρ*_0_ and *ρ*_300K_ exhibit sudden step-like increments (black arrows) at the pressures near 5.9 and 7.5 GPa.

To understand the evolution of electronic orders under external pressure, we have conducted temperature-dependent *ρ* measurements at pressures up to 12.9 GPa using a diamond anvil cell (Figure 2a,b). In the pressure regime starting from 0.5 GPa, we observe a clear *r* upturn around 318 K associated with the CDW1 ordering (a blue arrow). With further increase in pressure, the resistivity upturn is progressively weakened and its location is shifted to lower temperatures. Following a convention in the literature [23], we determine *T*_CDW1_ at each pressure as the peak temperature in the *d* ln(*ρ*)/*d*(1/*T*) curves (Figure 2c, blue arrows). As a result, *T*_CDW1_, located at 333 K at ambient pressure, systematically decreases to 40 K at 5.9 GPa. At *P* above 6.0 GPa, the peak associated with the CDW1 in the *d* ln(*ρ*)/*d*(1/*T*) curves could not be identified.

With a further increase in *P*, we find that another kink in the *d*ln(*ρ*)/*d*(1/*T*) curves emerges from 7.5 GPa and remains until ~11.3 GPa (red arrows in Figure 2d). Those kinks are linked to a weak resistivity drop, as illustrated in Figure 2b (red circles). Previous studies on the pristine CuTe have similarly found a small positive peak in the *dρ*/*dT* curves [11]; it was assigned as the onset of the second CDW order (CDW2), based on the observation of the amplitudon mode from Raman scattering measurements [12]. As the peak feature in the *dρ*/*dT* curves can appear as a dip in the *d*ln(*ρ*)/*d*(1/*T*) curve, we attribute the dip in the *d*ln(*ρ*)/*d*(1/*T*) curves to the onset of CDW2 order in Cu_0.984_Te.

It is found in Figure 2d that the determined *T*_CDW2_ from the dip of the *d*ln(*ρ*)/*d*(1/*T*) curves exhibits non-monotonous changes with pressure; it decreases from 176 K at 7.5 GPa to 170 K at 8.2 GPa, and again increases to 203 K at 11.3 GPa. This behavior is in contrast to that found in a pristine CuTe, where *T*_CDW2_, determined from the peak in the *dρ*/*dT* curve, continuously decreases with an increase in *P*; *T*_CDW2_ ≈ 204 K at 7.5 GPa decreases linearly down to *T*_CDW2_ ≈ 173 K at 10.1 GPa [12]. Note that the dip feature in the *d*ln(*r*)/*d*(1/*T*) curve becomes progressively weakened with further *P* increase and finally disappears at 12.9 GPa. We also confirmed that the pressure-dependent evolutions of both *T*_CDW1_ and *T*_CDW2_, as revealed in the *d*ln(*ρ*)/*d*(1/*T*) curves of Cu_0.984_Te (Figure 2d), align well with those found in the *dρ*/*dT* curves (Figure S5).

At low temperatures, the drop of *r*, implying the onset of superconducting transition, appears between 5.2 and 8.2 GPa (Figure 2e). The onset temperature of the superconducting transition (*T*_C_) is determined by a crossing temperature from the two linear extrapolated lines (dashed lines in Figure 2e). As a result, *T*_C_ reaches a maximum of 3.2 K at 5.6 GPa and gradually decreases with an increase in *P*. Note that this value is significantly higher than the maximum *T*_C_ = 2.3 K realized at *P* ≈ 5.7 GPa in CuTe [12].

As depicted in Figure 2f, we also find that both residual resistivity (*ρ***_0_**) and resistivity at 300 K (*ρ*_300K_) remain small at *P* ≤ ~6 GPa (a black arrow), where the vulcanite structure is dominant. On the other hand, at *P* ≥ ~9 GPa, where the *r*-CuTe structure is dominant, both *r***_0_** and *ρ*_300K_ reach the highest value. In an intermediate regime of 6.7 ≤ *P* ≤ 8.3 GPa, both *r***_0_** and *ρ*_300K_ remain in the intermediate values and exhibit gradual increments. At this intermediate *P* regime, the Raman scattering results exhibit evidence of structural coexistence in Cu_0.984_Te.

D. Structural phase transition and the emergence of CDW2

The emergence of CDW2 in Cu_0.984_Te and CuTe occurs in a different manner. In the Cu_0.984_Te, *T*_CDW2_ first appears at 176 K and at *P* = 7.5 GPa inside the pressure range where the *r*-CuTe structure coexists with the *v*-CuTe structure (i.e., 6.7 ≤ *P* ≤ 8.3 GPa). With the stabilization of the *r*-CuTe structure (*P* ≥ 8.3 GPa), *T*_CDW2_ decreases to 170 K, and increases again to 203 K as *P* increases to 11.3 GPa. In contrast, the pristine CuTe exhibits a sudden appearance of the CDW2 phase at 204 K at *P* ≥ 6.7 GPa in the *v*-CuTe structure, and *T*_CDW2_ decreases monotonically with increasing pressure [11,12]. It was previously argued that the CDW2 phase in CuTe may stem from electronic correlation effects induced by four hole pockets originating from Te *p_z_* orbitals, because there is no phonon softening or diverging electronic susceptibilities to support either electron–phonon coupling scenario or Fermi-surface nesting picture, respectively [12]. However, there is no concrete evidence in the theoretical calculations to support the scenario [12].

**Figure 3:**
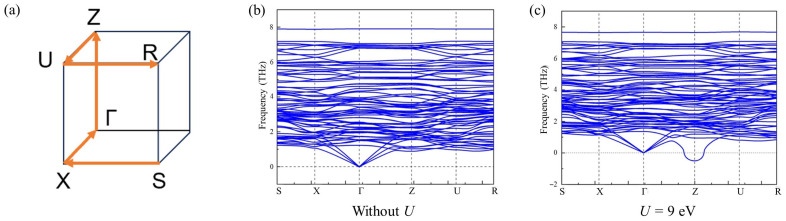
The result of the phonon band calculation on the Cu deficient Cu_11_Te_12_ at 10 GPa, which is constrained to have the *r*-CuTe structure. (**a**) Bulk Brillouin for the normal phase of the *r*-CuTe with high-symmetry points labeled. The phonon band structures of the *r*-CuTe (**b**) without and (**c**) with Coulomb interaction (*U* = 9 eV).

To better understand the origin of stabilizing the CDW2 phase in Cu_0.984_Te, we have thus calculated phonon band structures of the *r*-CuTe with Cu deficiency, using the Cu_11_Te_12_ composition at 10 GPa (Figure 3). For this, the initial atomic positions of the r-CuTe structure, as provided in the supplementary data as a cif file, were used to have Cu deficiency in the Cu sites. Then, at 10 GPa, the atomic positions of the *r*-CuTe structure with Cu deficiency have been relaxed. Since Coulomb interaction can affect the emergence of CDW2, as argued in CuTe [24], calculations were performed for the cases with or without Coulomb interaction (*U*) for Cu 3*d* orbitals. Without the Coulomb interaction, there was no structural instability. However, the results reveal a negative phonon mode near the *Z* point when the Coulomb interaction *U* = 9 eV is included. When *U* is systematically increased, it is found that the phonon mode softening was systematically increased at the Z point (See Figure S6). Our independent calculations of the phonon band structure for the pristine CuTe (*v*-CuTe structure) for both with (*U* = 9 eV) and without (*U* = 0) Coulomb interaction, did not result in any phonon anomaly (See Figure S7). However, when the chemical potential of the CuTe electronic structure has been shifted toward the hole-doped side, the effect of increasing *U* has also resulted in a similar negative phonon mode at a different momentum position (Figure S7). These results suggest that both Cu deficiency, making hole-doping, and increased Coulomb interactions should be important in creating the CDW2 phase in the high-pressure region of Cu_0.984_Te.

E. Magnetotransport measurements under high pressure

**Figure 4:**
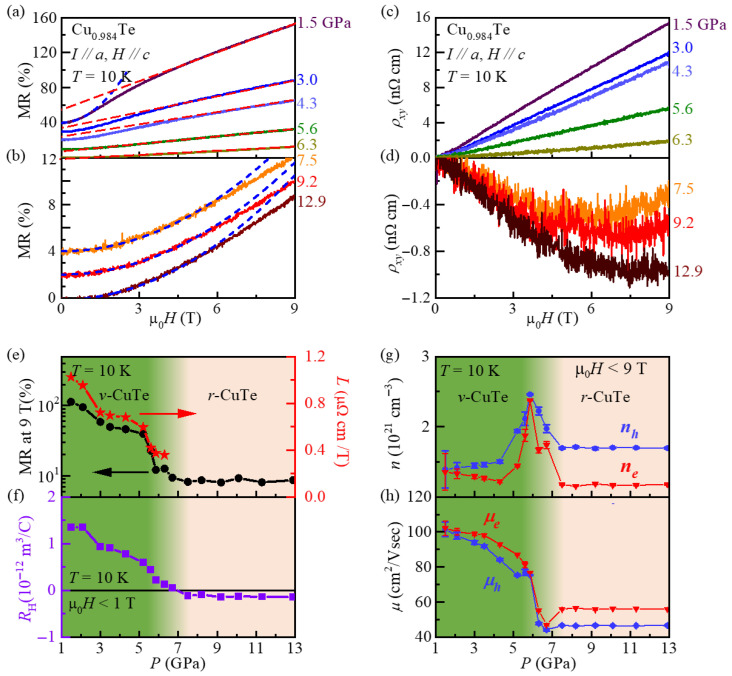
(**a**) Magneto-resistance (MR) data for *P* ≤ 6.3 GPa at 10 K. The red dashed lines represent the linear MR region observed above 3 T, which is established to emerge within the pressure range of having the CDW1 order as a ground state. The blue dashed line represents the quadratic MR region observed below 2 T in MR data for *P* = 1.5 GPa. The data is shifted by a constant (10%) for clarity. (**b**) The MR data for *P* ≥ 7.5 GPa at 10 K. The blue dashed lines represent the quadratic MR region observed below 6 T. The data is shifted by a constant (2%) for clarity. (**c**) Hall resistivity data *ρ_xy_* at 10K for *P* ≤ 6.3 GPa. (**d**) *ρ_xy_* at 10K for *P* ≥ 7.5 GPa. (**e**) The MR value at 9 T (black circles) and the linear slope *L* (*ρ*(*H*) = *ρ*_0_ + μ_0_*L*|*H*|) extracted from the *ρ*(*H*) curve at μ_0_*H* ≥ ~3 T (red stars). (**f**) Pressure dependence of the Hall coefficient (*R*_H_) at 10K and μ_0_*H* < ~1 T. (**g**) Pressure-dependence of hole- and electron-carrier densities (*n_h_* and *n_e_*) and (**h**) their mobilities (*μ_h_* and *μ_e_*) as obtained by the analyses of *ρ_xy_* by a two-band model.

To monitor the evolution of the electronic structure with pressure, we conducted magnetoresistance (MR) and Hall effects measurements at various pressures. Figure 4a shows the MR curves, Δ*ρ*(*H*)/*ρ*(0) × 100 = (*ρ*(*H*) − *ρ*(0))/*ρ*(0) × 100 at 10 K, and at *P* ≥ 1.5 GPa. For 1.5 ≤ *P* ≤ 5.6 GPa, where CDW1 is stabilized, the MR at 10 K is particularly large, reaching 10–110% at 9 T. Moreover, at this *P* regime, MR shows a crossover from *H-*quadratic at μ_0_*H* ≤ 2 T (dashed blue line) to linear dependence above μ_0_*H* ≥ ~3 T (dashed red guidelines). As *P* increases from 1.5 to 5.6 GPa, MR at 9 T decreases from 110 to 10%, accompanied by similar decreases in the linear slope (red dashed lines). Therefore, the large MR and the linear slope proportional to the MR value should be attributed to the characteristic features of the CDW1 state.

For *P* > 6.3 GPa and at 10 K (Figure 4b), where the CDW2 state in the *r*-CuTe structure is dominantly stabilized, the MR values become less than 10% at m_0_*H* = 9 T; they are clearly smaller than those in the lower pressure regime. Moreover, the field-dependence of MR, being proportional to ρ(H), exhibits mostly quadratic behavior, i.e., $\rho (H)\propto H^{\beta }$, with *β* ≈ 2 at μ_0_*H* ≤ ~6 T (dashed blue lines). Detailed analyses of the dρ(*H*)/dμ_0_*H* curves (Figure S8) are also consistent with such quadratic *H* dependence in the region where the CDW2 state in the *r*-CuTe structure is realized.

Numerous materials with CDW order have often exhibited large linear MR below their *T*_CDW_s [25]. In those cases, the CDW transition can create small electron or hole pockets with sharp curvature, called “sharp corners” in the momentum space. Electrons traveling along these sharply curved trajectories experience abrupt momentum changes, thus strongly influencing the resistivity values. Under magnetic fields, the electrons experience the Lorentz force, causing them to follow the trajectories with a cyclotron frequency proportional to *H*. This means that as *H* increases, the electrons pass through the trajectory more quickly and thus increase the probability of interaction with the sharp corners. As a result, the number of electrons interacting with such sharp corners can increase linearly with *H*, explaining how the CDW state can lead to the linear MR.

Based on the hypothesis that the linear MR in Cu_0.984_Te (Figure 4a) is also caused by the sharp corners in the momentum space as formed inside the CDW1 state [10], we try to extract the slope *L* predicted by the formula of *ρ*(*H*) = *ρ*_0_ + μ_0_*L*|*H*|. Figure 4e shows the resultant *L* extracted from the MR data (Figure 4a, μ_0_*H* ≥ 3 T) at each pressure; the MR value at μ_0_*H* = 9 T at 10 K is also plotted together. It is found that as *P* increases, both *L* and MR at 9 T systematically decrease up to 5.9 GPa, at which the CDW1 state is nearly suppressed to become *T*_CDW1_ = 40 K. At *P* = 6.3 GPa, the MR exhibits only linear *H*-dependence down to ~2 K. With further increase in *P* ≥ 6.3 GPa (Figure 4b), linear MR behavior disappears (thus *L* vanishes) and quadratic *H* dependence becomes dominant in the *ρ*(*H*) curves as well, shown in the three representative curves from 7.5 to 12.9 GPa. Moreover, the MR values at 9 T become saturated at ~10%. These results suggest that the reconstructed electronic structure formed by the CDW1 state produces sharp corners in the momentum space to result in the large linear MR. Moreover, upon pressure being increased to enter the CDW2 state, the sharp corners may have almost disappeared.

Figure 4c,d show the Hall resistivity *r**_xy_*** at 10 K measured at various *P* ≤ 6.3 and ≥7.5 GPa, respectively; the evolution of the Hall coefficient *R*_H_ extracted at the curves below 1 T is also summarized in Figure 4f. At *P* ≤ ~5.9 GPa, where the CDW1 order is stabilized, the positive *R*_H_ values (Figure 4f) systematically decrease with an increase in *P*, indicating that effective carrier density increases with the gradual decrease in the electronic gap inside the CDW1 state. However, even at *P* ≥ 6.3 GPa, where the CDW1 state is no longer stabilized at a finite temperature, *R*_H_ keeps decreasing to become negative at *P* = 7.5 GPa, from which nearly the same negative value is maintained up to *P* = 12.9 GPa. In addition, followed by the sign change of *R*_H_ near 7.5 GPa, *ρ_xy_* starts to exhibit nonlinear *H* dependence in the pressure region of ~7.5 ≤ *P* ≤ 12.9 GPa (Figure 4d). As *R*_H_ values (also *ρ_xy_* behaviors) are similar at 7.5 ≤ *P* ≤ 12.9 GPa, irrespective of the phase boundaries of CDW2 (7.5 ≤ *P* ≤ 11.3 GPa), it is expected that the *r*-CuTe structure, not the CDW2 state, mainly determines the electronic structures.

To analyze the *H* dependence of *ρ**_xy_*** under pressure, we used a two-band model with one-hole and one-electron bands [26,27]:

$$\rho_{xy}=\frac{B}{e}\frac{{(n}_{h}{\mu_{h}}^{2}-n_{e}{\mu_{e}}^{2})+(n_{h}-n_{e}){\mu_{h}}^{2}{\mu_{e}}^{2}B^{2}}{{\left(n_{h}\mu_{h}+n_{e}\mu_{e}\right)}^{2}+{{(n}_{h}-n_{e})}^{2}{\mu_{h}}^{2}{\mu_{e}}^{2}B^{2}}$$

Here, *n_h_* (*n_e_*) represents the hole (electron) density, *μ_h_* (*μ_e_*) is the hole (electron) mobility, *e* is one electron charge, and *B* is the magnetic induction. This model was applied to fit the *r**_xy_*** curve at each pressure (see Figure S9), and the parameters *n_h_*, *n_e_*, *μ_h_*, and *μ_e_* obtained from the fit are summarized in Figure 4g,h. It is found that at ~4.3 ≤ *P* ≤ 7.5 GPa, both *n_h_* and *n_e_* are sharply increased, forming maximum values at the critical pressure of ~5.9 GPa, at which a quantum critical point of the CDW1 state is expected to exist. Simultaneously, both *μ_h_* and *μ_e_* exhibit a nearly two-fold decrease from *P* ≈ 3.5 to 6.7 GPa and a slight increase at 7.5 GPa, thus forming a minimum at 6.7 GPa. At *P* ≥ ~7.5 GPa, *μ_h_* and *μ_e_* remain nearly constant. These observations suggest that a continuous gap closing of the CDW1 state by *P* increases up to ~5.9 GPa and associated critical fluctuation of charge amplitude might cause the increase in carrier densities and the decrease in carrier mobilities [28]. This aligns with the diminishing linear MR as *P* approaches ~5.9 GPa, showing that the suppression of the CDW1 order is significantly affecting the transport properties. Furthermore, under the CDW1 order, the electron- and hole-carrier densities, as well as the mobilities, take comparable magnitudes, suggesting a nearly compensated state. Such a condition, likely induced by the Fermi-surface reconstruction under the CDW1 state, can account for the observed large and linear MR [29,30,31].

When a single CDW state is collapsed within the same crystal structure, thereby closing the CDW gap, it is common to observe an increase in the carrier densities outside the CDW state [32]. However, our results indicate that near the structure coexistence region of the *v*- and *r*-CuTe (~6.7 ≤ *P* ≤ 8.3 GPa), the *n_h_* and *n_e_* show abrupt decreases from 6.3 GPa, while *μ_h_* and *μ_e_* increase slightly. This suggests that, albeit having the gap closing of the CDW1 state, the creation of different structural phases may have resulted in the abrupt reduction in carrier densities. In addition, possible extra-scattering of carriers at the structural domain walls, if any, may be negligible as compared with the effect of critical fluctuation of the CDW1 order (both CDW amplitude and phase). Furthermore, at *P* ≥ 7.5 GPa, where the CDW2 state is stabilized, the decreasing behavior of *n_h_* and *n_e_* values is saturated to remain nearly the same, indicating that the electronic structure, as influenced by the CDW2 state in the *r*-CuTe phase, is less sensitive to the pressure variation.

## 4. Discussion

**Figure 5:**
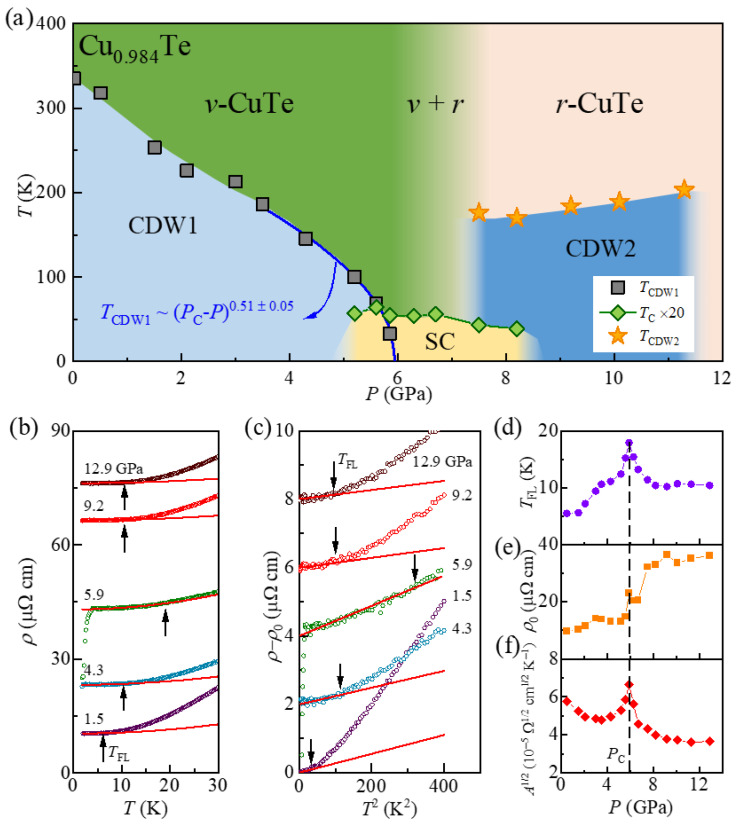
(**a**) The temperature-pressure (*T*-*P*) phase diagram of Cu_1-δ_Te (δ = 0.016). The blue dashed line indicates the suppression of CDW1 order. The blue solid line represents the power-law fitting (*T*_CDW1_~(*P*_C_-*P*) ^0.51 ± 0.05^ with *P*_C_ = 5.98 ± 0.05 GPa). In the region of green colors and the CDW1 state, the *v*-CuTe structural phase is stabilized, while in the pink region and the CDW2 region, a new structural phase of *r*-CuTe is stabilized. In the intermediate pressure range of ~6.7 ≤ *P* ≤ ~8.3 GPa, the two structural phases of the *v*-CuTe and *r*-CuTe are coexistent. (**b**) The *r* curves measured under pressures and low temperatures. The results obtained at the temperatures above the superconducting states are fitted by a quadratic power law *ρ* = *ρ*_0_ + *AT*^2^ (red solid lines). (**c**) The plots of (*ρ* − *ρ*_0_) vs. *T*^2^ for various pressures. The red lines are linear guidelines. The black arrows in (**b**,**c**) indicate the effective Fermi liquid temperature *T*_FL_. (**d**) The pressure dependence of *T*_FL_. (**e**,**f**) indicate the pressure-dependent evolution of the fitting parameters *ρ*_0_ and *A*^1/2^ obtained from the best fit to *ρ* = *ρ*_0_ + *AT*^2^ curves below *T*_FL_. The black dash indicates the critical pressure *P*_C_ obtained from the power law fitting described in Figure 5a.

Figure 5a summarizes the pressure-temperature phase diagram of Cu_0.984_Te, highlighting distinct regions of structural and electronic phases. Raman spectroscopy, resistivity, and magnetotransport data are used to construct the structural and electronic phase boundaries. The ambient *v*-CuTe structure is stabilized below 6.7 GPa, within which the CDW1 order emerges at low temperatures below *T*_CDW1_. As described in the Results, the *r*-CuTe structure coexists with the *v*-CuTe structure between 6.7 and 8.3 GPa. Above 8.3 GPa, the system fully transforms into the *r*-CuTe phase. The CDW2 emerges at ~176 K at *P* = 7.5 GPa, and *T*_CDW2_ is gradually enhanced up to 203 K with pressure increase up to 11.3 GPa. However, above 11.3 GPa, the experimental signature of the CDW2 state (i.e., dip in the *d*ln(*ρ*)/*d*(1/*T*) curves in Figure 2d) disappears.

We should note that the structural/electronic phase evolution of Cu_0.984_Te differs significantly from that of the pristine CuTe [12] in several aspects (Figure S1). Firstly, CDW1 in Cu_0.984_Te is continuously suppressed with pressure to reach *T*_CDW1_ = 40 K at 5.9 GPa, and the signals related to the CDW1 cannot be found in the transport data above 6.0 GPa. In contrast, the CDW1 state of the pristine CuTe persists up to 6.5 GPa and exhibits *T*_CDW1_~100 K near 6.5 GPa. Secondly, in the Cu_0.984_Te, we observe a clear structural phase transition into the *r*-CuTe structure and the emergence of the CDW2 state within the *r*-CuTe structure. This new structure was absent in the pristine CuTe, while the CDW2 state in CuTe has been found at 7.5 ≤ *P* ≤ 10.3 GPa. This indicates that the high-pressure structure is not an essential part of inducing the CDW2 instability. Thirdly, *T*_CDW2_ in Cu_0.984_Te increases with pressure from 170 K at 8.2 GPa to 203 K at 11.3 GPa. However, *T*_CDW2_ in CuTe decreased with pressure increase; it is found at 204 K at 7.5 GPa and 173 K at 10.3 GPa. Therefore, the slight deficiency of Cu significantly affects both the electronic and structural phase boundaries.

The CDW1 state in Cu_0.984_Te shows a decrease of *T*_CDW1_ with pressure increase. Indeed, we found that *T*_CDW1_ decreases rapidly in the low-*P* regime (*P* ≤ 3.5 GPa) (Figure 5a). On the other hand, above 3.5 GPa, where *P* approaches 6 GPa, *T*_CDW1_ follows a power-law behavior with $T_{CDW1}\propto {\left(\frac{P_{C}-P}{P_{C}}\right)}^{0.51\pm \;0.05}$ with *P*_C_~5.98 ± 0.05 GPa. This power-law behavior with the exponent 0.5 is a typical behavior expected in a mean-field type phase transition [33]. This characteristic mean field behavior has also been found in several materials near the quantum critical point (QCP) [28,33,34,35,36,37], including NbSe_3_ [34,35] and *β*-vanadium bronze [34,36], which belong to the Q1D materials exhibiting a CDW formation and a charge ordering, respectively. This emergence of power-law behavior in Cu_0.984_Te thus strongly suggests that it may undergo a pressure-induced quantum phase transition of the CDW state, and the CDW fluctuation near *P*_C_ plays a role in enhancing *T*_C_.

On the other hand, the continuous evolution of *T*_CDW1_ with pressure, following power-law behavior near *P*_C_ is distinctly different from that of the pristine CuTe, in which *T*_CDW1_ decreases almost linearly to 100 K at 6.5 GPa and disappears suddenly in a first-order manner above 6.5 GPa [12]. The different pressure responses suggest that Cu deficiency may allow the continuous decrease in the electronic CDW1 order parameter (i.e., CDW amplitude) to form the pressure-induced CDW-QCP at 5.98 GPa. For this, the structural transition expected in the CDW instability is decoupled so that the associated anomaly in the Te chain occurs at a higher pressure regime (>6.3 GPa). Note that this behavior is clearly different from that observed in a typical 1D-CDW system, in which a first-order CDW transition often occurs together due to the associated large entropy change involved in the lattice modulation.

To investigate the possible effect of charge fluctuations near *P*_C_ on transport phenomena, we have studied the pressure-dependent evolution of the Fermi-liquid behavior at low temperatures above *T*_C_ (Figure 5b,c). Due to the dominant electron-electron scattering of the Fermi-liquids at low temperatures [38], *ρ* exhibits a quadratic *T-*dependence of *ρ* = *ρ*_0_ + *AT*^2^, where *ρ*_0_ is a residual resistivity and *A* is the coefficient of the quadratic term [39]. In our data for *P* ≤ 1.5 GPa, the quadratic power-law successfully describes the *ρ* (*T*) behavior below~5 K, defined as *T*_FL_. Here, *T*_FL_ refers to a coherent temperature, below which a crossover from incoherent to coherent metallic state occurs, and the electron-electron scattering becomes dominant [38]. Our data thus indicate that the electron-electron scattering is dominant in the transport behavior at ambient and low-pressure region (*P* ≤ 1.5 GPa) below *T*_FL_, while the other factors, such as electron-phonon coupling, become more important above *T*_FL_.

It is found that the quadratic *T*-dependence persists across all the *P* ranges studied. Figure 5c shows the (*ρ_ab_* − *ρ*_0_) vs. *T*^2^ plots at various pressures, indicating that (*ρ_ab_* − *ρ*_0_) is linear with *T*^2^ below *T*_FL_. As *P* increases, *T*_FL_ rises from 5.6 K at 1.5 GPa to 17.9 K at 5.9 GPa, suggesting an enhancement of the effective Fermi-liquid energy scale near *P*_C_. However, after reaching a maximum at *P*~*P*_C_, *T*_FL_ drops to ~10 K at 8.2 GPa and remains nearly constant up to 12.9 GPa. (Figure 5d). This behavior can be interpreted as a modification of the Fermi surface by *P*. A larger Fermi surface implies that electrons can maintain a well-defined quasiparticle distribution over a wider *T* range, thereby possibly elevating *T*_FL_. Since the suppression of CDW1 often leads to an enlarged Fermi surface, the sharply enhanced *T*_FL_ near *P*_C_ should be attributed to the increased Fermi surface area. The observed *P*-dependent increase in the carrier densities up to 5.9 GPa (Figure 4g) is also consistent with this picture. However, sharp decreases in both carrier density and *T*_FL_ above 5.9 GPa, and their saturated behavior above 8.2 GPa, indicate that the full stabilization of the *r*-CuTe structure above *P* = 8.2 GPa and its resultant electronic band structure limits the increase in carrier density and Fermi surface.

In the Fermi liquid state, it is known that the Kadowaki-Woods relation, *A* = *α*_KW_ *γ*_0_^2^ [40], where *α*_KW_ is the Kadowaki-Woods ratio and *γ*_0_ is the Sommerfeld coefficient, is satisfied, resulting in the relation that *A*^1/2^ ∝ *γ*_0_. Moreover, *γ*_0_ can be expressed as *γ*_0_ = $\frac{1}{3}\pi^{2}k_{B}^{2}(1+\lambda_{ee}+\lambda_{ep})N(\varepsilon_{F})$ [41], where *k_B_* is the Boltzmann constant, $\lambda_{ee}$($\lambda_{ep}$) is the electron-electron (electron-phonon) coupling constant, and $N(\varepsilon_{F})$ is the electronic density of states at the Fermi level. Based on the experimental data summarized in Figure 5c, we have extracted the pressure*-*dependent evolution of *A*^1/2^ (Figure 5f). Below 3.0 GPa, *A*^1/2^ decreases from 5.75 × 10^−5^ Ω^1/2^cm^1/2^K^−1^ at 0.5 GPa to 4.79 × 10^−5^ Ω^1/2^cm^1/2^K^−1^ at 3.5 GPa. However, in the range 3.5–6.7 GPa near *P*_C_, *A*^1/2^ exhibits a sharp peak reaching a maximum of 6.65 × 10^−5^ Ω^1/2^cm^1/2^K^−1^ at 5.9 GPa. With increasing pressure, *A*^1/2^ gradually decreases again up to 12.9 GPa.

According to the Kadowaki-Woods relation, the evolution of *A*^1/2^ provides insights into changes in $\lambda_{ee}$, $\lambda_{ep}$, and $N(\varepsilon_{F})$. Since the new structural phase in Cu_0.984_Te appears above 6.7 GPa, $\lambda_{ep}$ likely does not change significantly near *P*_C_ [39]. Hence, the increased *A*^1/2^ near *P*_C_ seems to arise from the enhancement in $N(\varepsilon_{F})$ or $\lambda_{ee}$. As the effective Fermi surface area is expected to increase upon closing the CDW1 gap, the increasing trend of the *T*_FL_ and the carrier density across *P*_C_ can be reasonably understood. This may be accompanied by a gradual decrease of $N\left(\varepsilon_{F}\right)$, as the pressure evolution of the electronic structure can result in a decrease in the effective mass. In our experimental results, *A*^1/2^ indeed exhibits a gradual decrease in both low and high pressure regions except near *P*_C_, indicating that the gradual decrease can be explained by the overall decrease of $N\left(\varepsilon_{F}\right)$ with pressure. However, to explain the sharp increase of *A*^1/2^ near *P*_C_, where the critical fluctuations of the CDW order parameter are expected, we conjecture that the $\lambda_{ee}$ increase plays an important role. A similar sharp enhancement of *A*^1/2^ near *P*_C_ has also been observed recently in other systems with a CDW-QCP, such as 2*H*-Pd_0.05_TaSe_2_ [39]. However, it seems quite rare to find experimental evidence of such enhanced charge fluctuation at the critical pressure in a Q1D material. This seems to be realized because the electronic CDW transition is decoupled from the structural transition in Cu_0.984_Te due to the Cu deficiency.

The critical behavior of CDW1 in Cu_0.984_Te offers new insights into the relation between SC and CDW1. Near *P*_C_, *T*_C_ reaches its maximum value of 3.2 K in Cu_0.984_Te. This is much higher than that found in pristine CuTe [12]. The enhancement of *T*_C_ near *P*_C_ thus suggests that strong CDW1 fluctuation may also contribute to enhancing SC, similar to the recent observations in other systems with the CDW-QCP; Ti-doped CsV_3_Sb_5_ [9], Lu(Pt_1-*x*_Pd_x_)_2_In [42], and 2*H-*Pd_0.05_TaSe_2_ [39]. In contrast, CDW2 appears to have minimal effect on SC itself because it emerges above 7.5 GPa. Therefore, our observations here underscore the critical role of CDW1 fluctuation in enhancing superconducting pairing strength in Cu_0.984_Te, possibly through increased electron-electron interaction.

## 5. Conclusions

We have investigated the pressure-induced evolution of electronic and structural properties of Cu_0.984_Te. Our experimental findings reveal three distinct features that differ from the pristine CuTe. Firstly, CDW1 is completely suppressed near 6 GPa with *T*_CDW1_ approaching zero, accompanied by enhanced superconductivity with *T*_C_ reaching 3.2 K, significantly higher than the 2.3 K observed in the pristine CuTe. Secondly, a pressure-induced structural transition occurs above 6 GPa, where the system transforms from *v*-CuTe to *r*-CuTe through an extended coexistence region. Thirdly, CDW2 develops exclusively in the high-pressure *r*-CuTe phase above 7.5 GPa, with *T*_CDW2_ increasing under pressure up to 11.3 GPa, contrary to its behavior in pristine CuTe. First-principles calculations and Raman spectroscopy suggest that this unique CDW2 phase, resulting in unstable phonon dispersion, is stabilized by the two major effects, i.e., large Coulomb interaction and chemical doping effects. These findings show that slight Cu deficiency in CuTe fundamentally modifies structural and electronic phase boundaries, providing new insights into the role of charge density wave order on the stabilization of other electronic orders (i.e., superconductivity and another CDW order) and structural properties in low-dimensional materials.

## Data Availability

The original contributions presented in this study are included in the article/Supplementary Material. Further inquiries can be directed to the corresponding authors.

## Supplementary Materials

The following supporting information can be downloaded at: https://www.mdpi.com/article/10.3390/ma18215042/s1, Figure S1: Comparison of the phase diagrams between Cu_0.984_Te and pristine CuTe [12]. Figure S2: The Raman spectroscopy measurements results for Cu_1-**δ**_Te single crystals at 300 K and at ambient pressure. Figure S3: Capillary X-ray diffraction pattern of Cu_0.984_Te. Figure S4: The scanning electron microscope image of a Cu_1-δ_Te single crystal. Table S1: the 5-point average ratio of Cu and Te for five pieces of Cu_1-δ_Te from the same batch and their averages. Figure S5: The dρ/dT vs. T plots under pressure. Figure S6: The result of phonon band calculation on the Cu deficient Cu_11_Te_12_ at 10 GPa with different Coulomb interactions. Figure S7: The result of phonon band calculation on the pristine CuTe at 10 GPa with different Coulomb interactions. Figure S8: The field derivatives of resistivity at 10 K of a Cu_0.984_Te single crystal under pressure. Figure S9: the two-band fitting results of the Hall resistivity measured at (a) 3.5 GPa, (b) 6.3 GPa, and (c) 10.1 GPa. Cif file: the r-CuTe structure used for the phonon band calculation.
