# Supplementary material for: Enhanced Superconductivity near the Pressure-Tuned Quantum Critical Point of Charge-Density-Wave Order in Cu1-δTe (δ = 0.016)
Source: Materials (Basel). 2025 Nov 5;18(21):5042. doi: 10.3390/ma18215042 (PMC12608423; doi:10.3390/ma18215042)
Supplement: Supplementary file 1 [file materials-18-05042-s001.zip › materials-3916000-supplementary.pdf]

Supplemental Information of "Enhanced Superconductivity Near the Pressure-Tuned Quantum Critical Point of Charge-Density-Wave Order in $Cu_{1-\delta}Te$ (δ=0.016)"

Kwang-Tak Kim [1], Yeahan Sur [1], Ingyu Choi [1], Zifan Wang [2], Sangjin Kim [1], Dilip Bhoi [1], Duck Young Kim [2,*] and Kee Hoon Kim [1,3,*]

[1] Center for Novel States of Complex Materials Research, Department of Physics and Astronomy, Seoul National University, Seoul 08826, Republic of Korea
[2] Center for High Pressure Science and Technology Advanced Research (HPSTAR), Shanghai 201203, China
[3] Institute of Applied Physics, Department of Physics and Astronomy, Seoul National University, Seoul 08826, Republic of Korea

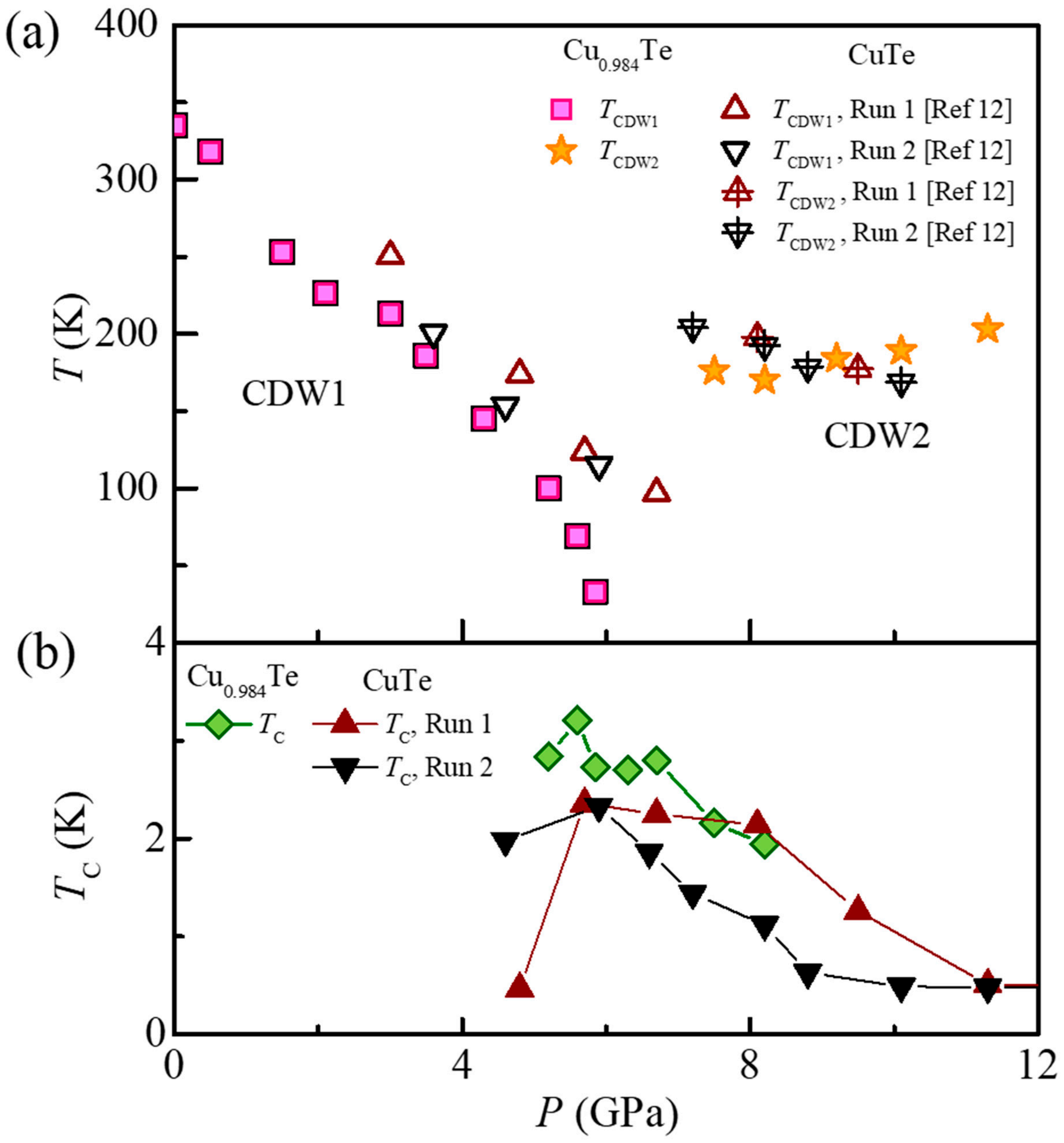

Figure S1. Comparison of the phase diagrams between $Cu_{0.984}Te$ and pristine CuTe[12] (a) CDW transition temperatures (b) T_C..

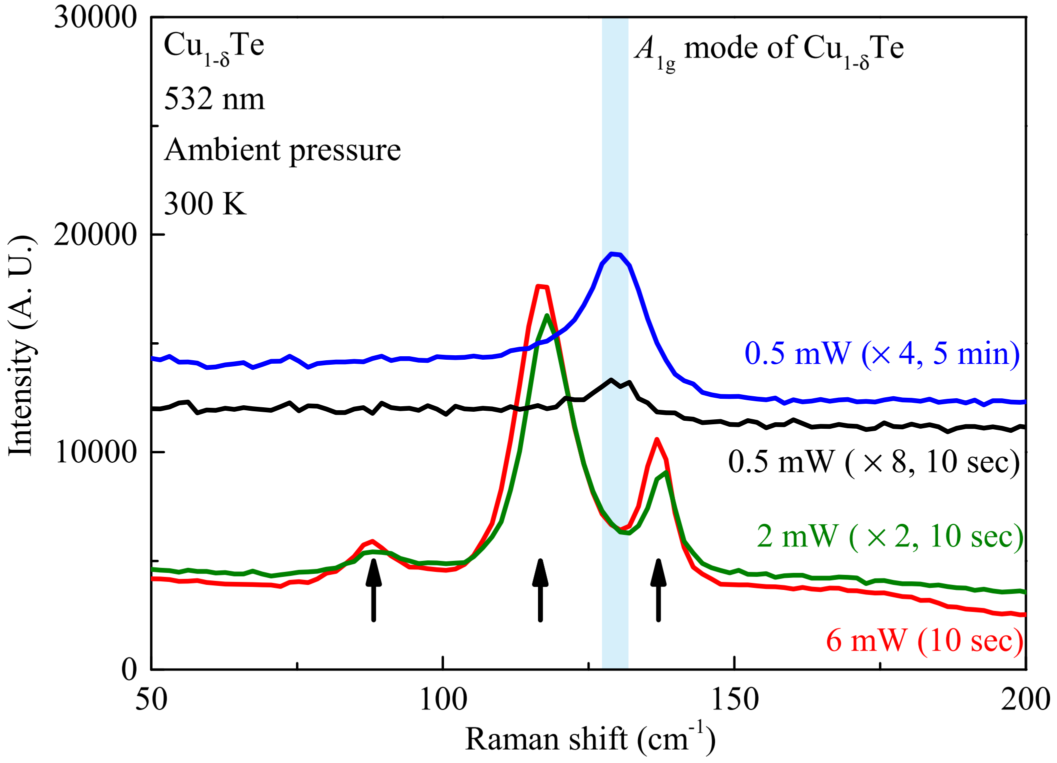

Figure S2. The Raman spectroscopy measurements results for $Cu_{1-\delta}Te$ single crystals at 300 K and at ambient pressure. The black arrows indicate the Raman modes induced by Te clusters [19].

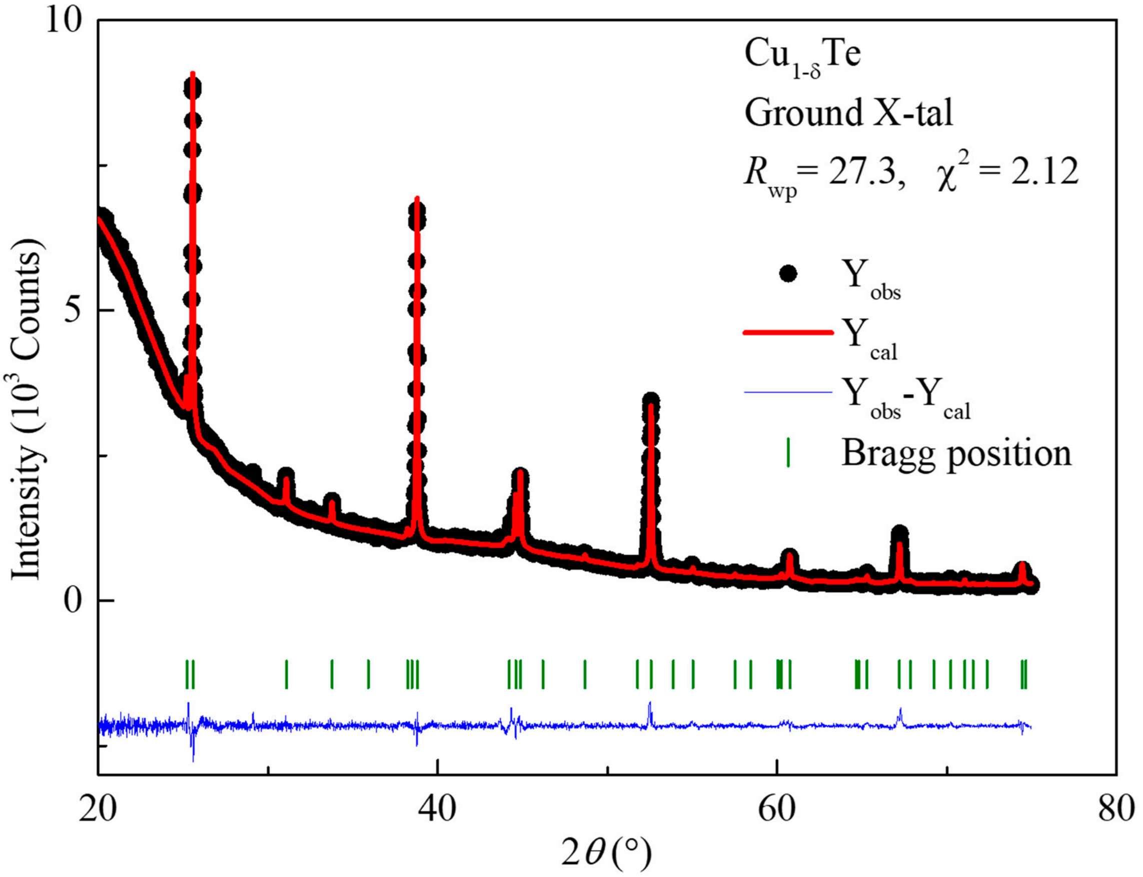

Figure S3. Capillary X-ray diffraction pattern of $Cu_{0.984}Te$. Capillary X-ray diffraction patterns (black dots, Y_{obs}) and the corresponding Rietveld refinement result (red line, Y_{cal}) with R_{wp} = 27.3 and χ^2 = 2.12 of a $Cu_{0.984}Te$ single crystal. The subtracted patterns (Y_{obs} - Y_{cal}) are shown as blue lines and the expected peak positions are shown as the green ticks.

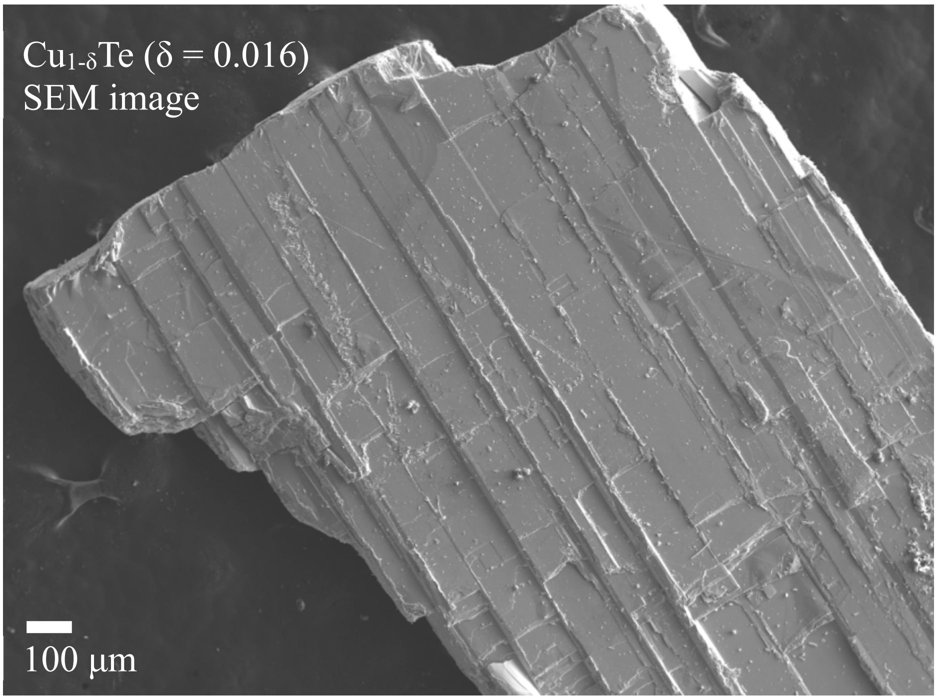

Figure S4. A scanning electron microscope (SEM) image of a $Cu_{1-\delta}Te$ (δ=0.016) single crystal. The relative ratio of Cu and Te is obtained by the analyses of wavelength-dispersive x-ray spectroscopy with a field emission electron probe microanalyzer. The horizontal bar shows the length scale of 100 μm.

Table S1. The relative ratio of Cu and Te in the 5 pieces of $Cu_{1-\delta}Te$ from the same batch, which were obtained by taking the averages over 5 different spots in each crystal surface.

	Piece 1	***Piece 2***	***Piece 3***	***Piece 4***	***Piece 5***	***Average***
Cu	49.497	49.762	49.665	49.598	49.431	49.591
Te	50.503	50.238	50.335	50.402	50.569	50.409
Std. dev.	0.191	0.085	0.158	0.280	0.314	

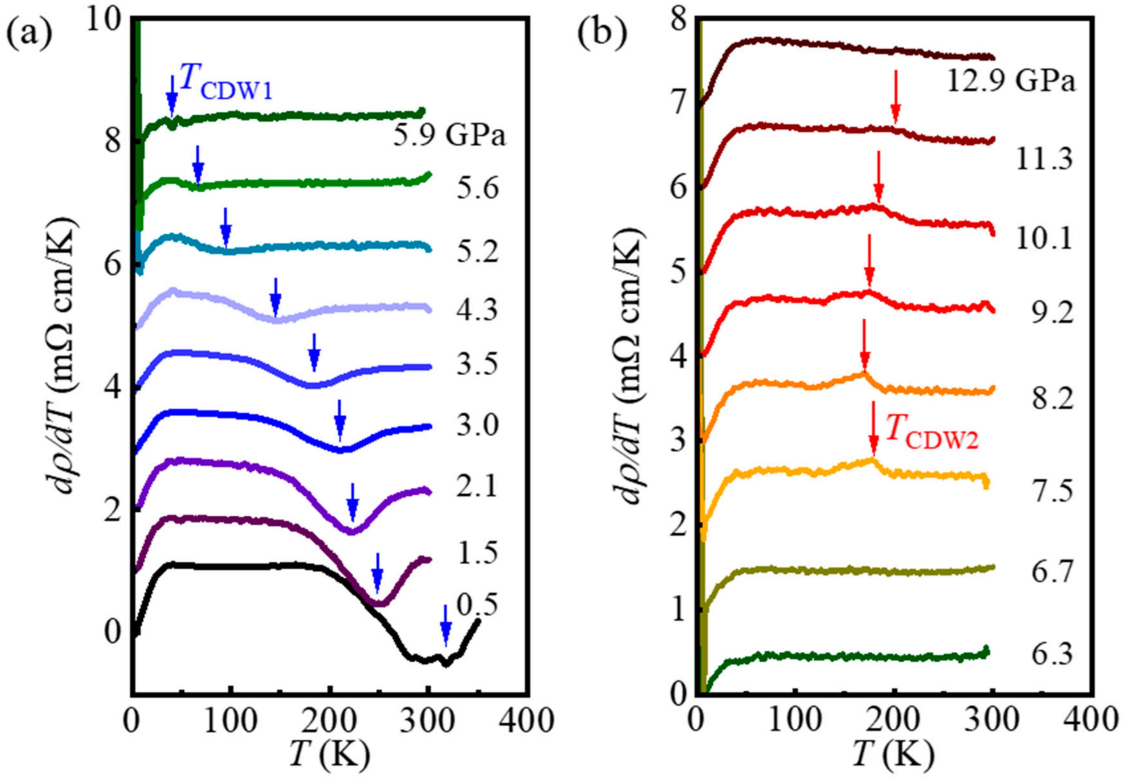

Figure S5. (a) $d\rho/dT$ vs. T plots of $Cu_{0.984}Te$ at $P \leq 5.9$ GPa. The blue arrows indicate the dips in each plot, representing T_{CDW1}. The data are shifted by a constant value for clarity. (b) $d\rho/dT$ plots for $P \geq 6.3$ GPa. The red arrows indicate the peaks due to stabilization of CDW2 phase in the new *r*- CuTe structure. The data are shifted by a constant value for clarity.

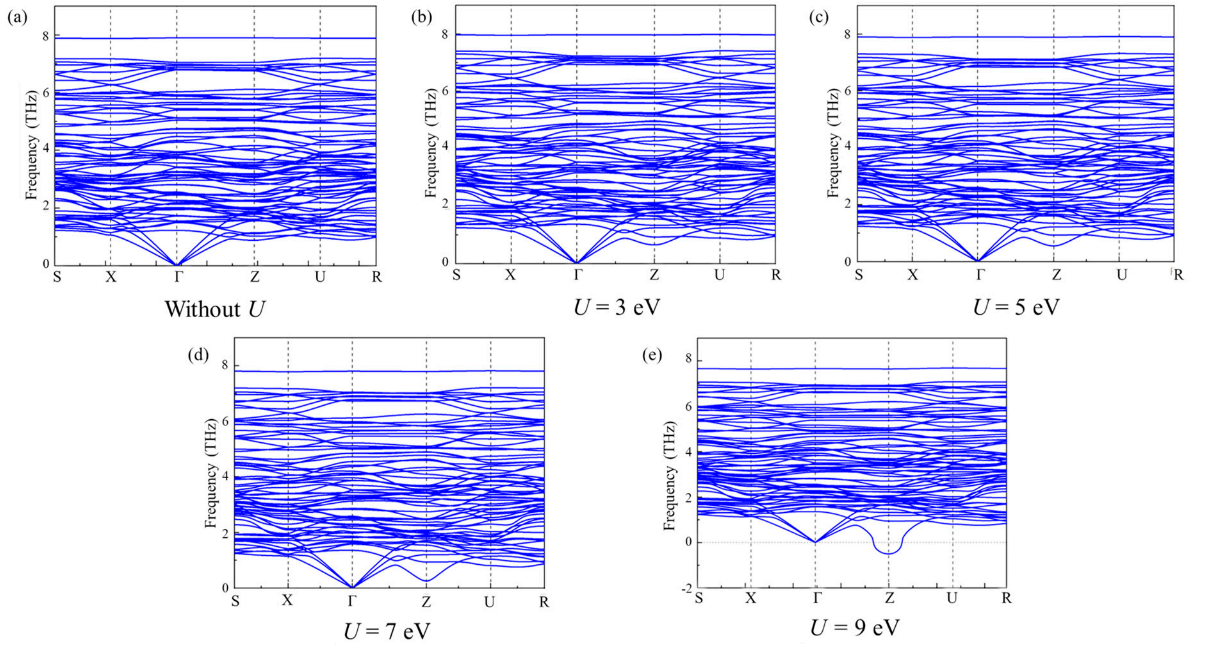

Figure S6. The result of phonon band calculation on the Cu deficient $Cu_{11}Te_{12}$ at 10 GPa with different Coulomb interaction (a) $U = 0$ eV, (b) $U = 3$ eV, (c) $U = 5$ eV, (d) $U = 7$ eV, and (e) $U = 9$ eV

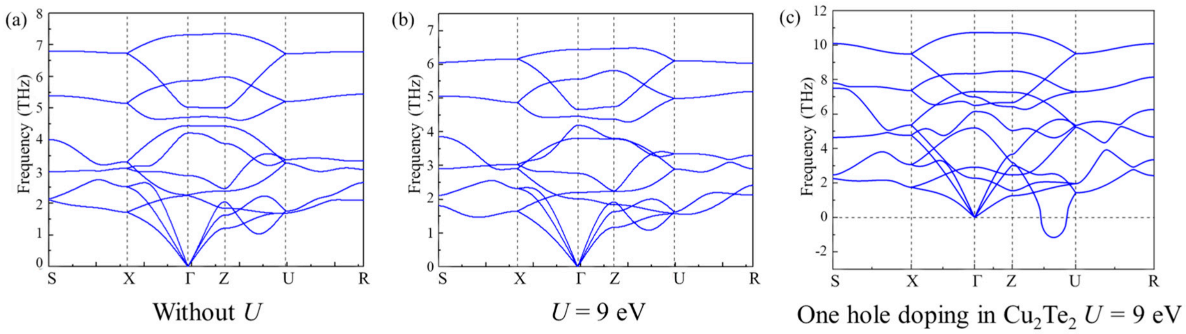

Figure S7. The result of phonon band calculation on the pristine CuTe at 10 GPa with different Coulomb interaction (a) U = 0 eV and (b) U = 9 eV (c) phonon band dispersion of the hole-doped pristine CuTe with coulomb interaction U = 9 eV.

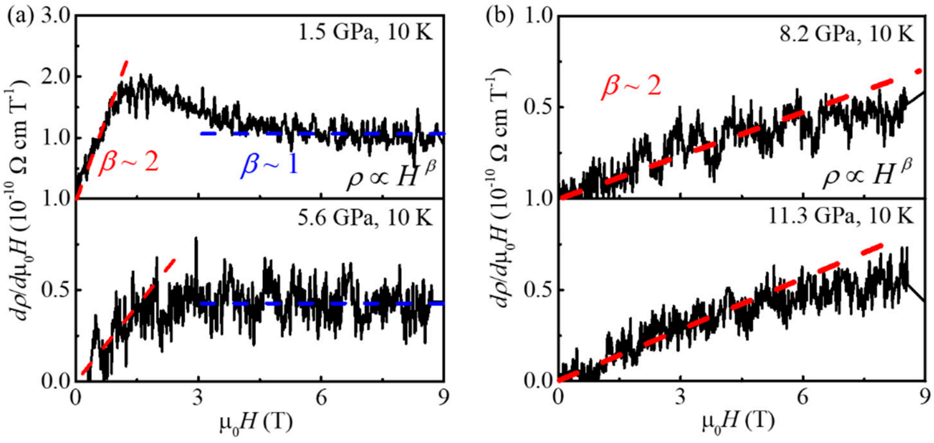

Figure S8. The field derivatives of resistivity at 10 K of a $Cu_{0.984}Te$ single crystal are plotted at pressures of (a) 1.5 and 5.6 GPa (b) 8.2 GPa and 11.3 GPa. The two sets of thepressures in (a) and (b) were chosen to investigate behavior before and after the suppression of CDW1 state, respectively. Based on the results, field-dependence of resistivity can be extracted as $\rho \propto H^{\beta}$. The red and blue dashed lines denote the regions with β=2 and β=1, respectively. In the pressure region where the CDW1 order is survived, it is found that a wide field region of $\mu_0 H$ > 3 T exhibits β=1; the $\rho \propto H$ behavior might be thus a characteristics of the reconstructed Fermi surface created by the CDW1 order.

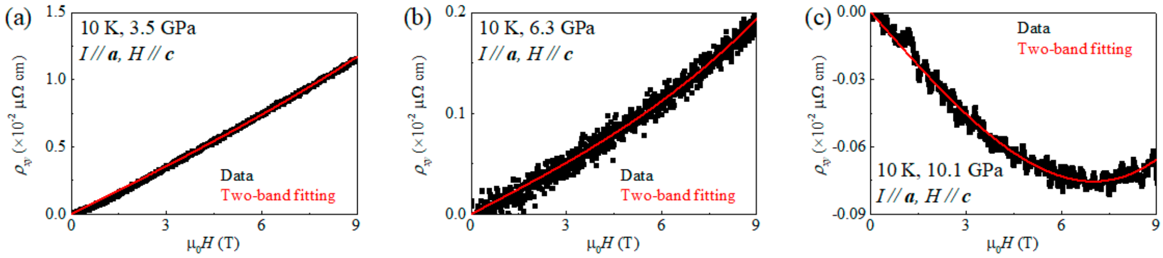

Figure S9. The two-band fitting results of the Hall resistivity measured at (a) 3.5 GPa, (b) 6.3 GPa, and (c) 10.1 GPa. The black squares indicate the measured data and the red solid line refers to the fitting curve for each case of (a)-(c).
